# Neurobiological predictors for clinical trajectories in fully remitted depressed patients

**DOI:** 10.1002/da.23108

**Published:** 2020-11-01

**Authors:** Thomas S. Blank, Bernhard M. Meyer, Ulrich Rabl, Paul Schögl, Marie‐Kathrin Wieser, Lukas Pezawas

**Affiliations:** ^1^ Department of Psychiatry and Psychotherapy Medical University of Vienna Austria

## Abstract

**Background:**

Serious long term health and economic detriment accompany residual depressive symptoms even in fully remitted depressed patients (rMDD). Neurobiological predictors for rMDD patients’ illness trajectory are absent.

**Methods:**

rMDD patients (*n* = 39, female = 26) underwent magnetic resonance imaging. Baseline analyses of brain structure via voxel‐based morphometry and brain function via functional connectivity (FC) at rest were correlated with changes in the Hamilton Depression Rating Scale between baseline and follow‐up, and incidence of a recurrent major depressive episode (MDE) within a 2‐year period.

**Results:**

Gray matter increases in default mode (DN) regions in the posterior cingulate cortex (PCC) and increased resting‐state FC within the DN both predicted change of depressive symptoms. Patients with recurrent MDE had larger bilateral nucleus accumbens and left insula volumes. Post hoc exploratory analysis of nucleus accumbens and insula conceptualized as part of the brain's reward circuit demonstrated reduced connectivity in patients with recurrent MDE.

**Conclusions:**

Higher DN connectivity and PCC volume coinciding with a more favorable course of symptoms suggest neural mechanisms of self‐recovery beyond the phase of active medical treatment. Alterations in the brain's reward circuit might be a starting point for designing maintenance treatments that prevent recurrent MDEs in rMDD patients.

## 1 INTRODUCTION

Most major depressive disorder (MDD) patients suffer from a chronic illness course. This not only entails long‐term health and economic detriment but also makes MDD the leading cause of years lived with disability (Zajecka et al., 2013). Patients typically experience relatively short durations of major depressive episodes (MDEs) but long and highly variable, subsequent illness trajectories. The rate of MDD patients who achieve full remission but continue to have residual symptoms is up to 80% (Nierenberg et al., 1999) often requiring long‐term maintenance treatment to forestall recurrent episodes (National Collaborating Centre for Mental Health (UK), 2010). While the majority of predictive imaging studies have explored determinants of remission from MDEs (Meyer et al., 2019; Phillips et al., 2015), stratification informed by the neurobiology of chronicity, specifically in terms of recurrent MDE and impairing residual symptoms, remains elusive.

The status of full remission commonly defined in clinical trials by a score of less than eight on the 17‐item Hamilton Rating Scale for Depression (HAMD) does, other than its nomenclature might suggest, seldom equate to an absence of symptoms. Primary remaining impairments are insomnia, anhedonia, fatigue, and cognitive problems, which is in contrast to the main diagnostic symptoms of an MDE (Pechtel et al., 2013). Clinically, the assessment of residual symptoms is of considerable importance since it facilitates the prediction of future MDEs (Zajecka et al., 2013). Moreover, the time span up to the next MDE is shortened more than threefold with even only minor residual symptoms present.

Standard treatment options for MDD are often insufficient to dispose of residual symptoms (Zajecka et al., 2013). The necessity to continue treatment even after the achievement of full remission led to specifically designed skill trainings to prevent recurrence with mindfulness‐based cognitive therapy (MBCT) as best‐practice intervention (National Collaborating Centre for Mental Health (UK), 2010).

Prospective, predictive neuroimaging designs for fully remitted MDD (rMDD) are missing, despite their major advantage over group‐comparisons: rMDD's phenotypic heterogeneity (Insel et al., 2010) can be harnessed for stratification and differential prediction of illness trajectories. Previous structural and functional magnetic resonance imaging (sMRI/fMRI) studies suggest that treatment failures are related to a dysfunction of the posterior (PCC) and anterior (ACC) cingulate cortex within the default network (DN; Meyer et al., 2019; Pizzagalli, 2011). Clinically, these findings were related to dysfunctional self‐referential cognition and rumination, so‐called sticky thoughts (Andrews‐Hanna et al., 2014), that persist independent of symptom improvement (Shilyansky et al., 2016) even after full remission (Bartova et al., 2015; Pizzagalli, 2011). Studies not only reaffirmed these cognitive traits known from concurrent MDEs in rMDD patients but also pointed to an often unrecognized loss of energy as a different, independent brain system (Zajecka et al., 2013). Lack of evidence prompts caution when translating these findings to maintenance therapy for rMDD. Still, the best‐evidence therapy, MBCT, appears to improve said cognitive symptoms together with normalizing the DN. Given the absence of valid animal models that approximate recurrence and residual, higher cognitive impairments, insights from neuroimaging could inform neurobiologically corroborated, tailored treatments that improve long‐term outcomes after full remission (Shilyansky et al., 2016).

Our decision to employ whole‐brain morphometry rested not only on the absence of predictive sMRI studies in rMDD, but more critically on its predictive capacity evidenced via meta‐analysis (Pizzagalli, 2011) and its low a priori assumptions compared to task‐based MRI. Second, we analysed resting‐state data to complement structural determinants on a functional network level, since resting‐state functional connectivity (RSFC) has been observed even between brain areas with little or absent structural connection (Damoiseaux & Greicius, 2009). Our prospective, predictive MRI study examined the two most important variables at this pivotal illness stage, residual symptoms change and recurrence operationalized by further MDE incidence. To our knowledge this is the first neuroimaging prediction study including rMDD patients. To this end, rMDD patients were clinically assessed and scanned (sMRI/fMRI), then clinically reassessed at a two‐year follow‐up. In an exploratory whole‐brain approach, we related sMRI data at baseline to residual symptom changes and recurrent MDE between assessments. Subsequently, RSFC was assessed to find brain network determinants of both clinical outcomes.

## 2 METHODS

### 2.1 Subjects and outcome variables

This prospective neuroimaging study was conducted at the Department of Psychiatry and Psychotherapy, Medical University of Vienna (MUV), Vienna, Austria. Fully remitted MDD patients were enrolled through public advertising. Written informed consent was obtained from all subjects. To achieve a comparable baseline and ensure the clinical significance of diagnosis, only patients who had received antidepressant medication or psychotherapy were recruited. Patients had discontinued any antidepressant treatment at least 3 months before study enrollment to rule out confounding mechanisms of heterogeneous psychopharmacological treatment. Study procedures were approved by the Ethics Committee of the MUV (Ethics Committee Number: 11/2008) in accordance with the Declaration of Helsinki. Recruitment, specifically inclusion and exclusion criteria, and assessments are described in full detail in (Bartova et al., 2015). Briefly, the baseline visit included psychiatric examination whereby diagnosis was ascertained via the German version of the Structured Clinical Interview for DSM‐IV Axis I disorders (Wittchen et al., 1997). The 17‐item HAMD was used to assess depression symptoms (Hamilton, 1986). Only subjects without any previous or current Axis I disorder were enrolled, whereby one MDE was mandatory for inclusion. Momentary rMDD status was ensured by requiring a 17‐item HAMD score of less than eight (Zajecka et al., 2013). Patients underwent MRI, including structural and resting state imaging sequences at baseline visit. This study extends the cross‐sectional study by Bartova et al. (2015) in that we reinvited patients from baseline visit to a 2‐year follow‐up psychiatric assessment.

Recurrent MDE was defined as a binary variable indicating whether at least one MDE between baseline visit and follow‐up occurred. We will also refer to those subjects as the MDE group in contrast to the no‐MDE group. HAMD change was defined as the difference between HAMD scores at baseline and follow‐up.

In our previous cross‐sectional study (Bartova et al., 2015), 78 rMDD subjects were recruited at the MUV. We were able to reinvite 39 patients for a follow‐up visit (Supporting Information, Section 2.1). Further 10 patients could be reached via telephone, when they were asked about MDEs between assessments. Twenty‐nine patients could not be included since they could not be reached via telephone or email. Since telephone interviews are of lower reliability than face to face clinical assessments, we chose to exclude these subjects from the main analysis due to quality concerns. Note, however, that an auxiliary analysis which included the 10 patients interviewed via telephone, yielded qualitatively equal regions (Supporting Information, Section 2.2) as those described in the main analysis below, thus corroborating our main findings. Moreover, a demographic comparison with the previous cross‐sectional study (Bartova et al., 2015) and the “telephone interview sample” indicated no relevant bias in our final sample (Supporting Information, Section 2.1, Table S1). Thirty RSFC datasets were available for analysis.

### 2.2 Voxel‐based morphometry analysis

Exact scanner specifications are described in the Supporting Information (Section Results, 3). Structural T1‐weighted images were preprocessed with the Computational Anatomy Toolbox (CAT12) for SPM (Gaser & Dahnke, 2016). Preprocessing steps included skull stripping, gray and white matter segmentation, and normalizing to a 1.5 mm structural MNI (Montreal Neurological Institute) template as provided by CAT12 after which spatial Gaussian kernel smoothing of 10 mm full width at half maximum was applied. Total intracranial volume was estimated for all subjects derived from gray matter segmentation in CAT12. SPM was used to compute group‐level correlations of gray matter volume relating to recurrent MDE and HAMD change, respectively, via an analysis of covariance (ANCOVA) model correcting for age, gender, and total intracranial volume. We applied a cluster forming threshold of *p*_uncorrected_ < .005 (Vasic et al., 2015). For multiple comparisons, FWE correction (*p*_FWE_ < .01) was used to allow for an additional Bonferroni correction by a factor of four to account for the two clinical variables and the twice one‐sided *t* testing, respectively.

### 2.3 Resting state preprocessing

Functional data were preprocessed using the CONN toolbox's standard preprocessing pipeline (Whitfield‐Gabrieli & Nieto‐Castanon, 2012). Briefly, steps included slice‐time correction, MNI normalization, and blurring at 10 mm FWHM. BOLD time series were band‐pass filtered to frequencies between 0.009 and 0.08 Hz. Movement parameters and nuisance signals from white matter and ventricles were regressed out.

### 2.4 Independent component analysis (ICA)

ICA with 20 components (Figure S1), a number reported to be effective in revealing large‐scale brain networks (Ray et al., 2013), was used to decompose resting‐state time series concatenated across subjects. Further CONN toolbox processing parameters (Nieto‐Castanon, 2020) were a reduction to the first 40 Singular Value Decomposition components, the FastICA G1/tanh algorithm (Hyvärinen, 1999) and Calhoun's GICA3 group ICA (Calhoun et al., 2001). GICA3 back‐projection results in spatially optimized subject‐specific fitted values which are a product of the original time course and spatial map.

**Figure 1:**
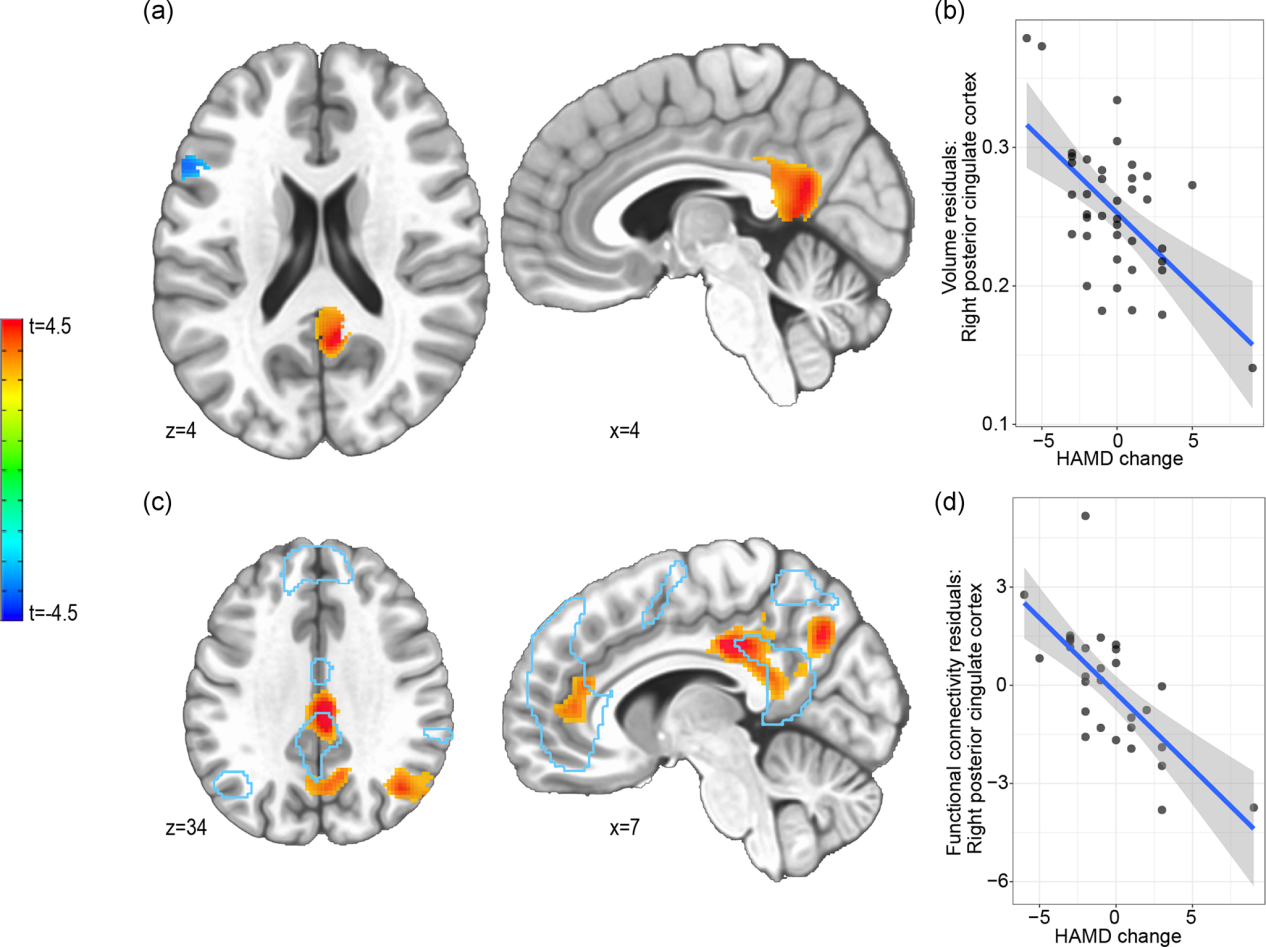
(a) Increased gray matter volume in the right PCC predicts symptom improvements as measured by HAMD scores (*p*_uncorrected_ < .005). (b) Scatter plot shows effects of symptom change on statistically significant right PCC cluster's voxel mean volume corrected for age, gender, and total intracranial volume. (c) Higher correlation of the PCC with the DN independent component was related to symptom improvements (*p*_uncorrected_ < .01). Blue contours outline the DN as identified via ICA. Note the spatial similarity of the DN contour and the VBM results in the PCC. (d) Scatter plot with mean DN component integration of the statistically significant PCC cluster corrected for age and gender. DN, default network; HAMD, Hamilton Depression Rating Scale; ICA, independent component analysis; PCC, posterior cingulate cortex

The component best fitting the DN was verified algorithmically by the CONN's match‐to‐template spatial correlation (Supporting Information, Section 4.1) and manually by visual inspection (Figure 1, blue contour). ANCOVA group‐level models were computed for DN component back‐projected values and the independent variables recurrent MDE and HAMD change, respectively, correcting for age and gender. A cluster forming threshold of *p*_uncorrected_ < .01 was followed by FWE correction of *p*_FWE_ < .01 to allow for an additional Bonferroni correction by a factor of two to account for the two variables.

### 2.5 Regions of interest (ROI) post hoc analyses

Since susceptibility to a future MDE was not reflected within the DN, but structural findings implicated alterations in the reward circuit, we ran a post hoc exploratory ROI‐to‐ROI RSFC analysis aiming to supplement structural findings with a description of functional interrelations. In addition to the reward circuitry (Cho et al., 2013), we also decided to test the central‐executive network (CEN; Seeley et al., 2007) due to its importance in emotion regulation (Etkin et al., 2015). Hence, ROIs selected from the CONN toolbox's atlas (Cho et al., 2013; Supporting Information, Section 5, Table S2) covered key regions consistently related to a suboptimal course in previous prediction studies (Meyer et al., 2019; Phillips et al., 2015). ROI‐to‐ROI group analysis was based on correlation matrices between the averaged BOLD time courses of ROI pairs (Nieto‐Castanon, 2020). The CONN toolbox allowed ANCOVA modeling, correcting for age and gender, on the temporal correlation coefficients of these matrices. An uncorrected single‐voxel threshold of *p*_uncorrected_ < .01 was followed by a FDR correction of *p*_uncorrected_ < .0025 to account for two tests.

## 3 RESULTS

### 3.1 Clinical characteristics

A total of 39 patients (female = 26, age mean = 26, *SD* = 5.6) had baseline and follow up data (Table 1). Ten patients (female = 9) reported an MDE after baseline evaluation. The median time between initial assessment and follow‐up (Ft) was 2.6 years. There was no significant difference in Ft between the MDE and no‐MDE group, nor a significant correlation with HAMD symptom change. Seven subjects in the no‐MDE group were reassessed before 1.5 years skewing the Ft‐by‐MDE group distribution. To rule out confounding influences of Ft, we recomputed all analyses excluding those seven patients, which, naturally, reduced statistical power but did not change the overall significance levels of our main findings (Supporting Information, Section 6).

**Table 1:** Continuous variables are presented as mean (standard deviation)

	No future MDE (*n* = 29)	Future MDE (*n* = 10)	*t/χ* ^2^	*p*
**Age, years**	26 (5.2)	25.8 (6.8)	0.1	0.92
**Females**	17	9	3.29	0.07
**Education, years**	12.8 (0.7)	12.4 (0.5)	1.5	0.14
**German vocabulary scale (WST)**	32.45 (6.72)	32.10 (2.84)	−0.23	0.82
**MDEs before 1st visit**	2.1 (2.9)	1.6 (1.3)	0.75	0.46
**HAMD 1st visit**	2 (1.8)	2.6 (1.7)	−0.89	0.39
**HAMD 2nd visit**	1.2 (1.4)	4.3 (4.5)	−2.1	0.06

Abbreviations: HAMD, Hamilton Depression Rating Scale; MDE, major depressive episode; WST, Wortschatztest (Schmidt & Metzler, 1992).

### 3.2 Reward circuit volume linked to recurrent MDE, PCC volume to symptom change

**Figure 2:**
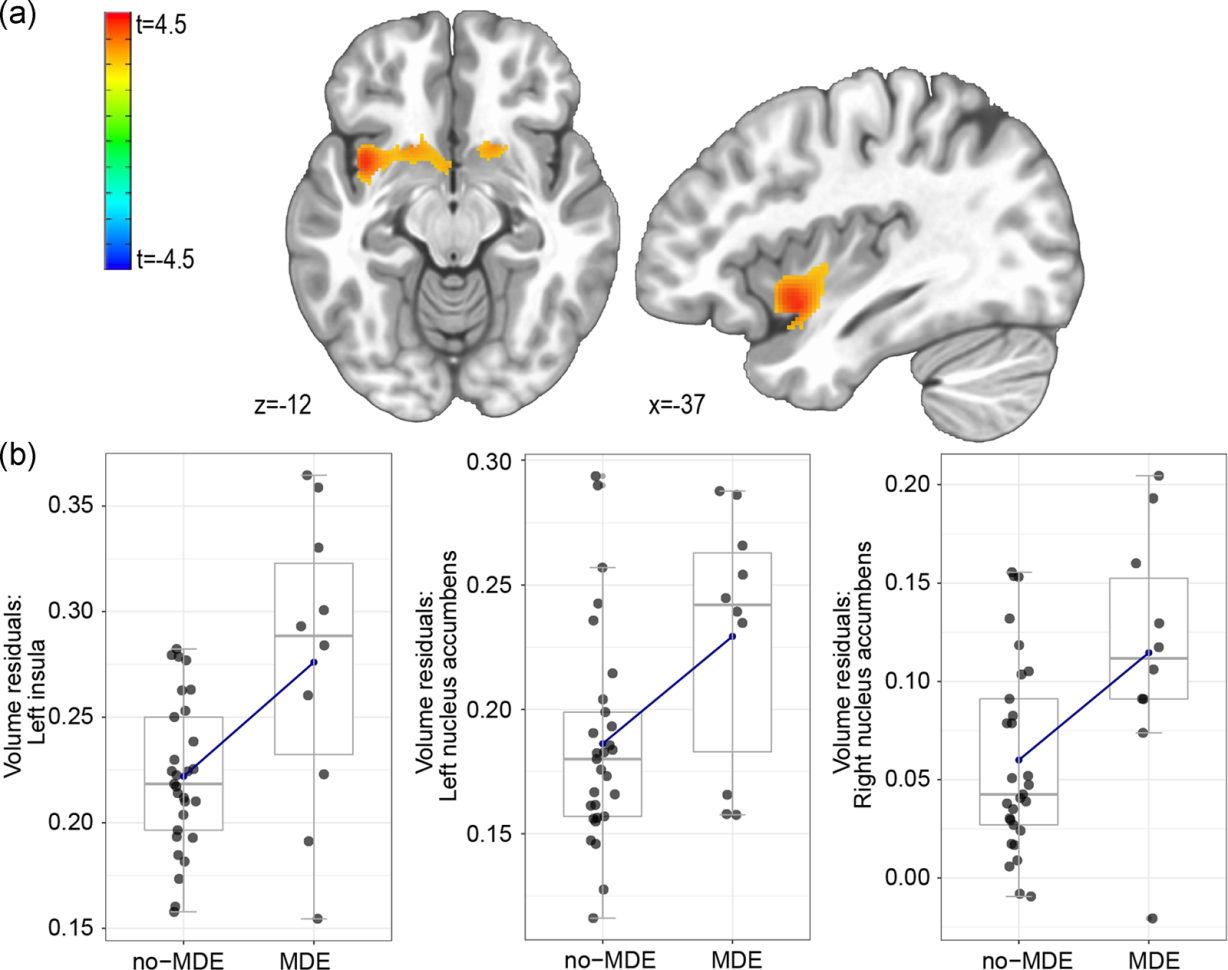
(a) Increased gray matter volume in the left insula and bilateral nucleus accumbens volumes predicts future recurrent MDE (*p*_uncorrected_ < .005). (b) Boxplots (blue line connecting the group means) indicate effects of recurrent MDE on statistically significant clusters’ voxel mean volumes corrected for age, gender, and total intracranial volume. MDE, major depressive episode

A whole‐brain VBM analysis was computed to detect predictors of recurrent MDEs and symptom change, respectively, explained via gray matter volume at baseline. Regarding correlation with recurrent MDE, VBM analysis (Table 2) predominantly exhibited increases in gray matter volume in bilateral nucleus accumbens (NAc) and left insula (Figure 2). Increases in gray matter volume in correlation with HAMD change were found in the right PCC (Figure 1 a,b). All significant regions up to a FWE corrected value of *p*_FWE_ < .05 are shown in full detail in (Supporting Information, Section 6, Table S3).

**Table 2:** Significant clusters (*p*_uncorrected_ < .005) of VBM analyses for variables HAMD change and recurrent MDE corrected at *p*_FWE_ < .01

VBM: MDE vs. no‐MDE	Region	Cluster size	*t* peak (df = 34)	*x, y, z* peak (MNI; LPI)	*t* cluster mean (df = 34)	Cohen's *d*
	Left anterior insula and left NAc	1370	4.2	−39, 6, −10	2.76	1.2
	Right NAc	477	4.41	32, 12, −22	3.59	1.42
	Left cuneus	161	3.4	−18, −58, −4	3.24	1.41
	Left lingual gyrus	218	−4.07	−18, −68, 32	−2.92	−1.16

VBM: HAMD change	Region	Cluster size	*t* peak (df = 34)	*x, y, z* peak (MNI; LPI)	*t* cluster mean (df = 34)	Kendall tau
	Right posterior cingulate cortex	1329	5.51	10, −48, 10	2.89	0.34
	Right superior frontal gyrus	553	4.56	21, 52, 6	3.97	0.46
	Right middle frontal gyrus	371	4.46	42, 28, 32	2.77	0.32
	Left inferior temporal gyrus	169	4.35	−56, −12, −22	2.47	0.29
	Left inferior frontal gyrus—pars triangularis	456	−3.91	−57, 18, 16	−2.69	−0.31
	Right postcentral gyrus	294	−3.8	62, −16, 42	−3.55	−0.41

RSFC: HAMD change	Region	Cluster size	*t* peak (df = 26)	*x, y, z* peak (MNI; LPI)	*t* cluster mean (df = 26)	Kendall tau
	Right posterior cingulate cortex	1338	5.62	−4, 28, 37	4.03	0.54
	Right angular gyrus	1037	4.97	−44, 60, 26	3.48	0.47
	Right medial frontal gyrus	388	4.23	−20, −58, −2	3.3	0.44

*Note*: Last three rows show RSFC clusters (*p*_uncorrected_ < .01) for ICA analysis with DN component, FWE corrected at *p*_FWE_ < .01. No significant RSFC clusters for recurrent MDE were observed.

Abbreviations: df, degrees of freedom; DN, default mode; FWE, family‐wise error; HAMD, Hamilton Depression Rating Scale; ICA, independent component analysis; LPI, orientation left‐posterior‐inferior; MDE, major depressive episode; MNI, Montreal Neurological Institute; NAc, nucleus accumbens; RSFC, resting state functional connectivity.

### 3.3 Strong RSFC in the DN predicts symptom improvement

We used a group ICA to distill a DN main component (Figure 1c, blue contour; Supporting Information, Section 4.1). Whole‐brain RSFC with this DN component was then correlated with the two clinical variables. HAMD change was linked to stronger within‐DN connectivity (*p*_uncorrected_ < .01, *p*_FWE_ < .01; Table 2; Figure 1c,d). No significant clusters associated with DN connectivity were found between the MDE group and the no‐MDE group.

### 3.4 Reduced RSFC in the reward circuit of the MDE group

We ran two post hoc exploratory ROI‐to‐ROI RSFC analyses aiming to supplement structural findings with a description of functional interrelations beyond the DN. For the reward circuit, a reduced RSFC between the right NAc and bilateral insula cortex (*p*_uncorrected_ < .01, *p*_FDR_ < .025) was found in the recurrent MDE group compared with the no‐MDE group. No significant relations between CEN regions could be found at the same thresholds.

## 4 DISCUSSION

This prospective study investigated the predictive value of sMRI and fMRI with respect to recurrence risk and residual symptoms in rMDD patients. First, increased gray matter volume in the left insula and bilateral NAc, as well as reduced RSFC among these regions, predicted future recurrent MDEs. Second, increased gray matter volume and stronger RSFC in the posterior DN, particularly the PCC, were indicators of future improvement of residual symptoms. Together, these multimodal findings relate two depression‐relevant brain networks (Bartova et al., 2015; Phillips et al., 2015) illustrating their importance for illness trajectory prediction in depression after full remission.

### 4.1 Recurrent MDE

Structural and functional correlates of recurrence exhibited increased gray matter volume (Figure 2) in and decreased functional connectivity between insular cortex and NAc. Both regions are major players within the brain's salience network and the reward circuit (Cho et al., 2013). During concurrent MDEs, previous research found reduced insula connectivity with subcortical limbic structures, such as the NAc (Park et al., 2019; Singh et al., 2018). Similarly, we observed that the recurrent MDE group had a weaker connectivitivity between bilateral insular cortices and the NAc compared with the no‐MDE group. Still, the directional interpretation is complex, given that the NAc's output is dominated by more than 95% inhibitory GABAergic neurons (Geldwert et al., 2006).

Anhedonia, which correlates with NAc alterations (Wacker et al., 2009), is one of the most persistent symptoms in rMDD after insomnia and fatigue (Nierenberg et al., 1999). Importantly, Pechtel et al. (2013) reported that anhedonia, specifically blunted responsiveness to rewards, persists even in rMDD patients and posed the question whether these deficits may predict future MDEs. Indeed, our results positively answer the question in terms of anhedonia's underlying neurobiology showing that alterations in the reward circuit are predictive of a future MDE. NAc‐insula coactivation has also been implicated in reward anticipation in the positive valence system (Cho et al., 2013), which is the major cognitive process complementary to loss anticipation in the Research Domain Criteria's (Insel et al., 2010) negative valence system. Failure in reinforced reward learning has been correlated with attenuated striatal activity during MDEs and in experiments with healthy controls (Santesso et al., 2008). One might relate this weaker functional coactivation to a reduced reward anticipation and consequently inability to experience rewarding situations.

Alterations in the reward circuit align with the neurocognitive perspective on depression (Roiser et al., 2012), which centers around negative information processing biases. This bias pertaining to the reward circuit in MDD might amplify negative feedback, but also reduces reward responsiveness and hampers decision making (Cho et al., 2013). On another reading, in the MDE group, the insula cortex might be less able to exert top‐down influence over striatal regions (Mayberg, 1997), which is supported by findings establishing RSFC directionality from insula to NAc (Cho et al., 2013). Weaker top‐down control might incapacitate the patient in realizing cognitive control over depressive emotional core symptoms like apathy and anhedonia. Our previous findings (Meyer et al., 2019) predicted recovery from acute depression based on alterations in the emotion regulation circuits (dlPFC‐amPFC; CEN). Though the role of the CEN could not be corroborated in the present study, the current finding related recurrence in rMDD to the reward circuit (Insula‐NAc). Both prediction studies underline the importance of an insufficient top‐down regulation between lateral cortical regions and the limbic system. These results support parallel neural circuitries (reward vs. emotion regulation) consistently tied to a suboptimal illness course (Phillips et al., 2015), which need to be specifically tested regarding neurocognitive risk factors, and also at different illness stages. Moreover, further research is needed to establish whether a specific anhedonia endophenotype (Drysdale et al., 2017; Pizzagalli et al., 2005) of rMDD is more likely to lead to future relapses and, conversely, whether rMDD patients with recurrent MDEs are more likely to be found among this subtype.

Most closely related to our structural findings, Zaremba et al. (2018) reported differing insula baseline volumes reaching from lower volume in healthy individuals to higher volume in MDD patients without future relapse up to MDD with relapse. The reason why this volume group difference in the insula appears to be more pronounced in our rMDD sample than in patients with a concurrent MDE might be due to a greater heterogeneity with respect to severity and symptom variance in concurrent MDE that could cover up volume differences. The gray matter volume increase in the MDE group in these regions (Figure 2) could possibly be due to a long term over‐compensatory effect of the underlying, weakened neural communication between insula and NAc.

### 4.2 HAMD symptom change

In our sample, within‐default‐network RSFC correlated with HAMD change (Figure 1c,d; Table 2) as did PCC volume (Figure 1a,b; Table 2), its structural counterpart Strikingly, there existed a remarkable alignment between structural and functional clusters. Two non‐mutually exclusive interpretations of these findings can be drawn.

Firstly, using resting state imaging in remitted young adults, one study found DN hyperconnectivity, specifically anterior cingulate to posterior cingulate cortex connectivity, inversely correlated to depression symptoms (Jacobs et al., 2014). This is in line with our findings in the sense that DN hyperconnectivity led to symptom improvement. Jacobs et al. proposed that DN hyperconnectivity in rMDD patients might be protective or even preventive of further MDEs. Although our study did not directly demonstrate the latter, it lends support to the protection hypothesis when interpreting the protective effect as HAMD scores improvement. Behaviorally, most accounts of DN hyperconnectivity in depression presume a link with unconstructive rumination (Jacobs et al., 2014). However, numerous studies highlight the adaptive consequences of repetitive thought such as coping with traumatic events, future planning, depression recovery and initiating health‐promoting actions (Watkins, 2008). Specifically the PCC‐amPFC core systems, the subsystem overlapping strongest with the DN component in this study (Supporting Information, Section 4.2, Figure S2), was previously associated with personally significant, emotional and introspective mental states (Andrews‐Hanna et al., 2010). Thus, DN hyperconnectivity rather than constituting a neurobiological marker of rMDD might be a biological, curative response to depression giving rise to adaptive self‐referential cognitive functions. Notably, the utilization of introspective cognition is a major feature of MBCT, the recommended therapy for rMDD patients (National Collaborating Centre for Mental Health (UK), 2010).

Second, reduced PCC volume in MDD has been reported in several studies (Caetano et al., 2006; Grieve et al., 2013). Caetano et al. depicted a gradual increase in PCC volume from concurrent MDE patients, to rMDD patients, up to healthy individuals. Our findings associating a larger PCC with future symptom improvement might portray a similar trend: on the route from rMDD to recovery, PCC volume increases might approximate the normal volume of healthy individuals, just as clinically rMDD patients’ HAMD scores approximate healthy individuals’ absence of symptoms. Ultimately, patients with larger DN volume and connectivity might be those whose potential for improvement and, consequently, route to normalization has yet to be accomplished. This hypothesis of the DN constituting a state marker that neurobiologically mimics a patient's momentary position on her recovery trajectory finds support through other studies that have associated the anterior DN as a trait‐like indicator and the posterior DN comprising the PCC as state‐like indicator in depression (Li et al., 2013).

### 4.3 Synthesis

Prediction of recurrent MDEs and symptom change were linked to differing regions structurally and functionally in rMDD patients. Each outcome measure's importance stems from its particular temporal properties along the illness course; whereas recurrent MDE reflects a singular event during a time period, HAMD symptom change constitutes the longitudinal difference in HAMD score between baseline and follow‐up visit. Correspondingly, while reward circuitry alterations predicted further MDEs, the DN appears to be not only important during a concurrent MDE (Kaiser et al., 2015; Meyer et al., 2019) but exerts long term influence even in relatively minor symptom changes in rMDD. The relative structural and functional separation of the positive and negative valence systems (Insel et al., 2010), DN and reward circuit, evidence the distinctness of both outcome variables. Specifically, posterior DN connectivity appears to implicate a neural state of insufficient remission likely to moderate following improvements of residual symptoms. In contrast, recurrent MDE findings in the reward circuit essentially constitute predictive states indicative of a future MDE. In view of treating subthreshold depressive symptoms, it might thus be necessary to target both systems; interventions aimed at the normalization of the DN to achieve stable remission, and restoration of reward anticipation to reduce risk of imminent recurrence even after full remission.

### 4.4 Clinical perspective

In line with our previous study (Meyer et al., 2019), the DN might provide a proxy for monitoring satisfactory depression recovery. This has, indeed, been observed for antidepressants, electroconvulsive therapy (Abbott et al., 2013) and transcranial magnetic stimulation (Liston et al., 2014) where DN hyperconnectivity predicts response to the respective intervention. The reward circuit and related anhedonia symptoms are particularly relevant for MDD patients with chronic illness courses and treatment‐resistant patients. Hence, the NAc within the mesolimbic dopaminergic system is known to constitute an important target of various forms of second‐line treatments including deep brain stimulation (Schlaepfer et al., 2008), D_2_ antagonists like amisulpride (Admon et al., 2017) or dopamine reuptake inhibitors like bupropion (Tomarken et al., 2004) that aim to counteract attenuated striatal activity. Beyond that, these compounds could be specifically important in the prophylactic treatment of rMDD patients, possibly achieving prevention of a future MDE by normalizing connectivity within the reward circuit.

The low proportion of male participants in the recurrence group might be related to lower help‐seeking behavior and hospital admission compared to female patients (Burcusa & Iacono, 2007). A meta‐analysis notes that women are more likely to suffer from a recurrent MDE than men, although the effect is heterogeneous and small (Bertschy et al., 2016).

### 4.5 Strengths and limitations

We were able to reinvite 39 follow‐up patients of our previous study (Bartova et al., 2015) transforming it into a prospective longitudinal study design. Nonetheless, the study would have benefitted from a larger sample, particularly regarding the recurrent MDE group, which was almost exclusively composed of women. Incorporating gender as a nuisance variable in our models did not compromise our findings. Gender main effects were not spatially related to our clinical findings. Still, due to the large proportion of females in the recurrent MDE group, recurrent MDE results warrant only limited applicability to men. We utilized several quality assurance steps implemented in the CONN toolbox including artifact and outlier detection. However, a limitation of the toolbox is that no reliability measures, like bootstrapping of ICA components, are available (Nieto‐Castanon, 2020). Concerning strengths, our study is the first rMDD prediction study describing this understudied but clinically highly relevant stage of the depressive illness course. Moreover, we combine structural and functional imaging modalities in a longitudinal design describing neurological underpinnings of two major clinical characteristics, recurrent MDE and symptom change.

## 5 CONCLUSION

Shifting research focus from patients with concurrent MDE to rMDD and acknowledging the debilitating influence of residual symptoms this longitudinal study started at a point of the illness course where most studies are ending. Our findings implicate that reward circuit and DN appear to be brain structural and functional predictors of recurrence and residual symptom change. The specific role of each circuit after remission exhibits mechanisms in the rMDD population that are more than a mere attenuation of a known MDD mechanism. DN hyperconnectivity, a robust feature of concurrent MDE, was associated with symptom change. This warrants the hypothesis that DN hyperconnectivity might be a self‐recovery process rather than a negative trait or state marker in rMDD, which coincides with PCC gray matter volume augmentation. Our study once more highlights the capacity of the DN to reflect the current state of depression in all stages of MDD. Moreover, recurrence was associated with the reward‐circuit, thus supporting maintenance therapies aiming to normalize reward responsiveness.

## CONFLICT OF INTERESTS

The authors declare that there are no conflict of interests.

## AUTHOR CONTRIBUTIONS

*Designed research*: Thomas S. Blank, Bernhard M. Meyer, Lukas Pezawas, and Ulrich Rabl. *Performed research (acquisition, analysis, and interpretation of data)*: Thomas S. Blank and Bernhard M. Meyer. *Wrote the paper*: Thomas S. Blank and Bernhard M. Meyer. *Critically revised the paper*: Lukas Pezawas, Ulrich Rabl, Paul Schögl, and Marie‐Kathrin Wieser. *Had full access to all of the data in the study and takes responsibility for the integrity of the data and the accuracy of the data analysis*: Lukas Pezawas.

## Supporting information

Supporting information.

## Data Availability

The data that support the findings of this study are available from the corresponding author upon reasonable request.
