# Supplementary material for: Neurobiological predictors for clinical trajectories in fully remitted depressed patients
Source: Depress Anxiety. 2020 Nov 1;38(4):447–55. doi: 10.1002/da.23108 (PMC8048641; doi:10.1002/da.23108)
Supplement: Supplementary file 1 — Supporting information. [file DA-38-447-s001.docx]

Supplement

# 1. Sample comparisons and re-analysis

## 1.1 Sample comparison

A demographic comparison of the three samples mentioned in the paper is provided in Table S1. The “Bartova sample” [(Bartova et al., 2015)](http://sciwheel.com/work/citation?ids=2683452&pre=&suf=&sa=0) includes all rMDD patients scanned at the first session. The “Telephone sample” is composed of those patients from the Bartova sample that could be contacted and interviewed for the follow-up assessment. However, ten subjects could only be interviewed via telephone, i.e., not in person, the subtraction of those yielding the “Final sample”.

|  | **Bartova sample** | **Telephone interview sample** | **Final sample** | **F / χ2** | **p** |
| --- | --- | --- | --- | --- | --- |
| n | 78 | 49 | 39 |  |  |
| Age, years | 26.7 (5.3) | 26.1 (5.4) | 26 (5.6) | 0.9 | 0.41 |
| Females (%) | 56% | 52% | 64% | 0.91 | 0.64 |
| Education, years | 12.5 (1.5) | 12.7 (0.9) | 12.7 (0.7) | 2.69 | 0.07 |
| German vocabulary scale (WST) | 33 (4.5) | 32.5 (5.5) | 32.4 (5.9) | 0.66 | 0.52 |
| MDE before 1st visit | 2.2 (2.4) | 1.9 (2.4) | 2 (2.6) | 0.59 | 0.56 |
| HAMD 1st visit | 1.7 (1.6) | 2.2 (2.5) | 2.2 (1.7) | 0.84 | 0.44 |
| Subjects with MDE between first and second assessment | NA | 11 | 10 | 0.12 | 0.73 |

Table S1. Shows a demographic comparison of the three samples mentioned in the paper. Continuous variables are presented as mean (standard deviation). Abbreviations: MDE, major depressive episode; HAMD, Hamilton Depression Rating Scale; WST, Wortschatztest [(Schmidt and Metzler, 1992)](http://sciwheel.com/work/citation?ids=7517715&pre=&suf=&sa=0)

Note that no test was significant at p<0.05. The small p-value for Education is due to a single outlier. Table S3 indicates no relevant difference between the previous cross-sectional study (Bartova sample), the “Telephone interview sample” or final sample. Only one person (male) of those interviewed via telephone only reported an MDE at follow-up.

## 1.2. Re-analysis for the telephone interview sample

Linear models were specified as follows:

grey_matter ~ MDE_group + age + gender + total_intracranial_volume

Including additional ten rMDD patients (total n=49) via telephone contact yielded with MDE_group as variable of interest for the left insula and left NAc cluster p_MDE_=.015 (t=2.54), and for the right NAc cluster p_MDE_=.025 (t=2.32). Thus, the MDE analysis’ main clusters remain statistically significant with regard to the MDE group. Exploratory RSFC analysis testing connectivity from right NAc to bilateral insular cortices remained trendwise significant (p_FDR_<.1) in the extended sample (n=37).

# 2. Scanner parameters

Magnetic resonance imaging (MRI) data were acquired on a 3 Tesla (3T) TIM Trio scanner equipped with a Siemens 12-channel head coil. They were visually inspected regarding data quality during and after scanning. Head movements were limited using foam pads and were quantitatively assessed and controlled for by using movement parameters. fMRI data were acquired via a phase-corrected blipped gradient echo, single-shot echo-planar imaging sequence (TE/TR=42/2000ms, 96 × 96 matrix, 210mm square FoV, 20 axial slices, slice thickness=4mm, slice gap=1mm) using an interleaved slice acquisition scheme. sMRI data were acquired with a 3D MPRAGE sequence (TR/TE=2300/4.21ms, 240x256x176mm FOV, flip angle 9 degrees, inversion time 900ms, voxel size of 1x1x1.1mm) during the same session as the fMRI data.

# 3. DN main component

## 3.1 Match-to-template fit

To verify which of the 20 components (Figure S1) obtained from the group ICA best fitted the DN, we calculated a match-to-template spatial correlation using a DN template from a well-published ICA [(Shirer et al., 2012)](http://sciwheel.com/work/citation?ids=972656&pre=&suf=&sa=0). The three best matching templates to Shirer et al.’s main component [(Shirer et al., 2012; Figure 1A, first slice)](http://sciwheel.com/work/citation?ids=972656&pre=&suf=%3B%20Figure%201A%2C%20first%20slice&sa=0) were ICA5 (r=0.39), ICA12 (r=0.16), and ICA2 (r=0.16). Thus ICA5, which we have called the DN main component, was chosen.


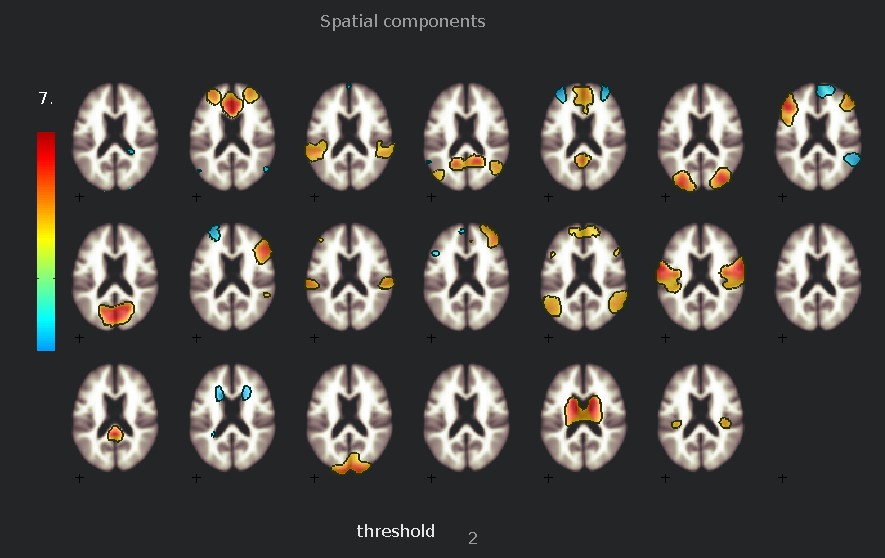


Figure S1. Group ICA components calculated and depicted via the CONN toolbox. ICA5, i.e., the fifth slice in the first row had the best fit to Shirer’s template [(Shirer et al., 2012; Figure 1A, first slice)](http://sciwheel.com/work/citation?ids=972656&pre=&suf=%3B%20Figure%201A%2C%20first%20slice&sa=0). Abbreviations: ICA, independent component analysis; CONN, connectivity toolbox

## 3.2 Subnetworks

A more precise impression of how the main DN component, retrieved via group ICA, is constituted can be gained by comparing it to well-established DN sub-networks. Figure S2 contrasts our DN component with three sub-networks, the PCC-aMPFC Core, the dMPFC Subsystem and the MTL (medial temporal lobe) Subsystem [(Andrews-Hanna et al., 2010; Table S1)](http://sciwheel.com/work/citation?ids=355969&pre=&suf=%3B%20Table%20S1&sa=0). The PCC-aMPFC core is fully captured by our DN component, while the dMPFC and MTL sub-systems partially overlap with missing parts e.g. in the parahippocampus (PHG, sagittal) and the temporoparietal junction (TPJ, axial).


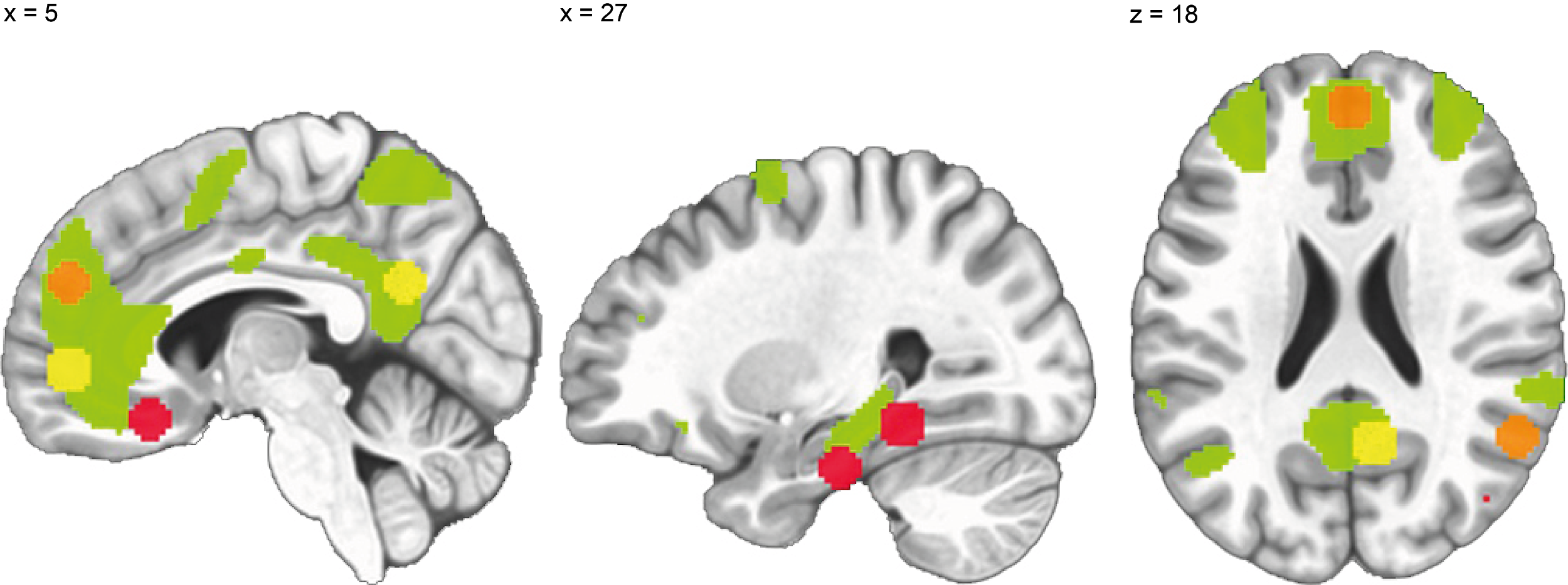


Figure S2. Depicts two sagittal and one coronal slice with DN component derived from the group ICA in green. Yellow, red, and orange 8 mm spheres (as in Andrews-Hanna et al.’s paper) indicate the PCC-aMPFC Core, the dMPFC Subsystem and the MTL (medial temporal lobe) Subsystem, respectively [(Andrews-Hanna et al., 2010)](http://sciwheel.com/work/citation?ids=355969&pre=&suf=&sa=0). Abbreviations: PCC, posterior cingulate cortex; aMPFC, anterior medial prefrontal cortex; dorsal medial prefrontal cortex; MTL, medial temporal lobe; DN, default network

# 4. ROI-to-ROI *post-hoc* analyses

To complement structural findings pertaining to relapse in the reward system by functional data, the *post-hoc* ROI-to-ROI analysis included the brain regions in Table S2. The selection of reward associated regions was based on Cho et al.’s description of the reward circuit [(2013)](http://sciwheel.com/work/citation?ids=382894&pre=&suf=&sa=1). For the CEN, regions were drawn from Seeley et al. [(2007)](http://sciwheel.com/work/citation?ids=148811&pre=&suf=&sa=1). Brain regions’ anatomy corresponds to the Harvard-Oxford atlas [(Jenkinson et al., 2012)](http://sciwheel.com/work/citation?ids=23618&pre=&suf=&sa=0) definitions for cortical and subcortical structural brain regions as implemented in CONN [(Whitfield-Gabrieli and Ford, 2012)](http://sciwheel.com/work/citation?ids=83175&pre=&suf=&sa=0).

| **Reward Circuit** | **Regions** | **Central executive network** | **Regions** |
| --- | --- | --- | --- |
|  | Anterior insula (left) |  | Posterior parietal cortex (left) |
|  | Anterior insula (right) |  | Posterior parietal cortex (right) |
|  | Nucleus accumbens (left) |  | Lateral prefrontal cortex (left) |
|  | Nucleus accumbens (right) |  | Lateral prefrontal cortex (right) |
|  | Thalamus (left) |  |  |
|  | Thalamus (right) |  |  |

Table S2. Shows the regions included in the *post-hoc* ROI-to-ROI analyses for the reward circuit and CEN.

# 5. Follow-up time (Ft) re-analysis

Since seven subjects were re-assessed at follow-up prior to 1.5 years, we re-analysed without those subjects (n=32). Linear models specified as in (1) above substituting MDE_group as variable of interest yielded statistically significant results for the left insula and left NAc cluster (p_MDE_=.0023; t=3.37), and for the right NAc cluster (p_MDE_=.0039; t=3.16). Replacing the variable of interest with HAMD change in (1), we obtained p_HAMD_=.0015 (t=3.54) for the PCC cluster. As in the main analysis, no significant clusters (p_FWE_<.01) associated with DN connectivity were found between the MDEbetween MDE and no-MDE group corrected for age and gender in the reduced sample (n=23). Exploratory *post-hoc* RSFC analysis testing connectivity from right NAc to bilateral insular cortices remained significant (p_FDR_<.05). Finally, re-analysis of DN connectivity in relation to HAMD change in the reduced sample yielded the same within-DN results when thresholded at p_uncorrected_<.05.

# 6. Full main results table

| **VBM: MDE vs. no-MDE** | **region** | **cluster size** | **t peak (df=34)** | **x y z peak (MNI; LPI)** | **t cluster mean (df=34)** | **Cohen's d** |
| --- | --- | --- | --- | --- | --- | --- |
|  | Left Anterior Insula & Left NAc | 1370** | 4.2 | -39 6 -10 | 2.8 | 1.2 |
|  | Right NAc | 477** | 4.4 | 32 12 -22 | 3.6 | 1.42 |
|  | Left Cuneus | 161** | 3.4 | -18 -58 -4 | 3.2 | 1.41 |
|  | Left Lingual Gyrus | 218** | -4.1 | -18 -68 32 | -2.9 | -1.16 |
|  | Left Calcerine Gyrus | 151* | -3.9 | -15 -80 -2 | -4.6 | -1.36 |
|  | Right Middle Frontal Gyrus | 131* | -3.9 | 27 18 58 | -3.6 | -1.21 |
| **VBM: HAMD change** | **region** | **cluster size** | **t peak (df=34)** | **x y z peak (MNI; LPI)** | **t cluster mean (df=34)** | **Kendall tau** |
|  | Right Posterior Cingulate Cortex | 1329** | 5.5 | 10 -48 10 | 2.9 | 0.34 |
|  | Right Superior Frontal Gyrus | 553** | 4.6 | 21 52 6 | 4 | 0.46 |
|  | Right Middle Frontal Gyrus | 371** | 4.5 | 42 28 32 | 2.8 | 0.32 |
|  | Left Inferior Temporal Gyrus | 169** | 4.3 | -56 -12 -22 | 2.5 | 0.29 |
|  | Right Inferior Occipital Gyrus | 136* | 3.4 | 27 -100 -3 | 3.5 | 0.41 |
|  | Left Middle Orbital Gyrus | 135* | 4.8 | -2 52 -8 | 2.4 | 0.28 |
|  | Right Middle Temporal Gyrus | 129* | 4.9 | 46 -70 -6 | 3.3 | 0.39 |
|  | Left Inferior Frontal Gyrus - Pars Triangularis | 456** | -3.9 | -57 18 16 | -2.7 | -0.31 |
|  | Right Postcentral Gyrus | 294** | -3.8 | 62 -16 42 | -3.5 | -0.41 |
|  | Right Lingual Gyrus | 139* | -3.4 | 24 -58 -8 | -2.5 | -0.29 |
| **RSFC: HAMD change** | **region** | **cluster size** | **t peak (df=26)** | **x y z peak (MNI; LPI)** | **t cluster mean (df=26)** | **Kendall tau** |
|  | Right posterior cingulate cortex | 1338** | 5.6 | -4 28 37 | 4 | 0.54 |
|  | Right angular gyrus | 1037** | 5 | -44 60 26 | 3.5 | 0.47 |
|  | Right medial frontal gyrus | 388** | 4.2 | -20 -58 -2 | 3.3 | 0.44 |

Table S3. Significant clusters (p_uncorrected_<.005) of VBM analyses for variables HAMD change and recurrence FWE corrected at p_FWE_<.01 (**) and p_FWE_<.05 (*). Last three entries show RSFC clusters (p_uncorrected_<0.01) for ICA analysis with DN component, FWE corrected at p_FWE_<.01. Abbreviations: df, degrees of freedom; HAMD, Hamilton Depression Rating Scale; LPI, orientation left-posterior-inferior; df, degrees of freedom; MDE, major depressive episode; MNI, standard brain Montreal Neurological Institute; NAc, nucleus accumbens; RSFC, resting state functional connectivity

# References

[Andrews-Hanna, J.R., Reidler, J.S., Sepulcre, J., Poulin, R., and Buckner, R.L. (2010). Functional-anatomic fractionation of the brain’s default network. Neuron *65*, 550–562.](http://sciwheel.com/work/bibliography/355969)

[Bartova, L., Meyer, B.M., Diers, K., Rabl, U., Scharinger, C., Popovic, A., Pail, G., Kalcher, K., Boubela, R.N., Huemer, J., et al. (2015). Reduced default mode network suppression during a working memory task in remitted major depression. J. Psychiatr. Res. *64*, 9–18.](http://sciwheel.com/work/bibliography/2683452)

[Cho, Y.T., Fromm, S., Guyer, A.E., Detloff, A., Pine, D.S., Fudge, J.L., and Ernst, M. (2013). Nucleus accumbens, thalamus and insula connectivity during incentive anticipation in typical adults and adolescents. Neuroimage *66*, 508–521.](http://sciwheel.com/work/bibliography/382894)

[Jenkinson, M., Beckmann, C.F., Behrens, T.E., Woolrich, M.W., and Smith, S.M. (2012). FSL. Neuroimage *62*, 782–790.](http://sciwheel.com/work/bibliography/23618)

[Schmidt, K.H., and Metzler, P. (1992). WST-Wortschatztest.](http://sciwheel.com/work/bibliography/7517715)

[Seeley, W.W., Menon, V., Schatzberg, A.F., Keller, J., Glover, G.H., Kenna, H., Reiss, A.L., and Greicius, M.D. (2007). Dissociable intrinsic connectivity networks for salience processing and executive control. J. Neurosci. *27*, 2349–2356.](http://sciwheel.com/work/bibliography/148811)

[Shirer, W.R., Ryali, S., Rykhlevskaia, E., Menon, V., and Greicius, M.D. (2012). Decoding subject-driven cognitive states with whole-brain connectivity patterns. Cereb. Cortex *22*, 158–165.](http://sciwheel.com/work/bibliography/972656)

[Whitfield-Gabrieli, S., and Ford, J.M. (2012). Default mode network activity and connectivity in psychopathology. Annu. Rev. Clin. Psychol. *8*, 49–76.](http://sciwheel.com/work/bibliography/83175)
